# Effect of Contact Lenses on Contrast Sensitivity under Various Lighting Conditions

**DOI:** 10.18502/jovr.v16i4.9742

**Published:** 2021-10-25

**Authors:** Monireh Mahjoob, Samira Heydarian

**Affiliations:** ^1^Health Promotion Research Center, Zahedan University of Medical Sciences, Zahedan, Iran; ^2^Department of Rehabilitation Sciences, School of Allied Medical Sciences, Mazandaran University of Medical Sciences, Sari, Iran

**Keywords:** Contact Lens, Contrast Sensitivity, Glare, Visual Acuity

## Abstract

**Purpose:**

To assess contrast sensitivity in clear and colored soft contact lenses under different lighting conditions.

**Methods:**

This study was performed on 34 medical students. Visual acuity was measured using a tumbling E chart at a distance of 6 m, and contrast sensitivity was determined by Pelli Robson chart at a distance of 1 m. These tests were repeated in mesopic (3 lux) and glare (2000 lux) conditions. Then, a clear contact lens was applied to one eye and a colored contact lens was applied to the other. After 2 hr, visual acuity and contrast sensitivity were measured for each individual. The results were compared with and without contact lenses under normal, mesopic, and glare conditions.

**Results:**

The mean refractive error was 0.44 ± 0.20 diopters. Repeated measures ANOVA showed a decline in contrast sensitivity with colored and clear contact lenses as compared to no-lens condition (*P* < 0.001). Additionally, lighting conditions had a significant impact on contrast sensitivity (*P* < 0.001); contrast sensitivity was lower in mesopic and glare conditions than under normal lighting condition.

**Conclusion:**

In addition to the drop in contrast sensitivity under unusual lighting conditions (e.g., glare and mesopic), wearing soft contact lenses can further reduce contrast sensitivity in different lighting conditions. Therefore, people who wear contact lenses should be aware of this reduction in visual performance in conditions like driving at night or in the fog.

##  INTRODUCTION

Contact lenses are gaining increasing popularity nowadays. Approximately 125 million people use contact lenses worldwide.^[1]^ Alongside its cosmetic aspect, the most important indication for using a contact lens is to correct refractive errors. Due to changing the color of the eyes, colored contact lenses are mainly used for cosmetic purposes, especially by women and young people.^[2]^

Among the various types of contact lenses, rigid gas permeable lenses (RGPs) are the least common and are fitted by about 2% due to the preference of practitioners and patients for soft contact lenses and the main reasons for this include the initial discomfort with rigid lenses and easier fitting of soft contact lenses.^[3]^ Silicone hydrogel contact lenses were initially developed for extended wear to eliminate hypoxia during overnight wear and they comprise 41% of all soft contact lenses fitted.^[4,5]^ Despite extensive studies on adverse symptoms with hydrogel and silicone hydrogel contact lenses, there is no evidence that silicone hydrogel contact lenses have significantly improved comfort compared to hydrogel contact lenses for daily wear.^[5,6]^ In addition, the incidence of microbial adverse events of the two types of soft contact lenses were found to be similar.^[5,7]^

One of the most significant issues in refractive correction is the quality of vision under different lighting conditions. Research findings suggest that contrast sensitivity (CS) tests show a more accurate assessment of quality of vision than visual acuity tests.^[8]^ People are also exposed to different lighting conditions in their daily lives, such as driving in foggy conditions or at night, which can affect their quality of vision.^[9,10]^ According to existing evidence, information obtained from CS tests under normal lighting conditions is not adequate.^[9,10]^ Therefore, measuring CS under different lighting conditions can determine people's vision quality more accurately.

Contrast of the retinal image can be reduced due to light scattering. Lens and cornea are two important sources of light scattering in the eye which are highly dependent on the age and opacity of the ocular media.^[11]^ Applying contact lenses, as an optical surface, can affect the rate of light scattering in the eye.^[12]^ Multifocal contact lenses can trigger visual problems like visual haloes, decreased CS, and fluctuating vision due to pupil size variations.^[13,14]^ Previous studies have reported different results for CS changes in single focal contact lenses.^[2,15,16]^ Wachler et al observed a drop in CS only at high spatial frequencies for clear contact lenses.^[17]^ However, some other studies have noted changes in CS occurring only in colored contact lenses.^[2,16,18]^ Considering the inconsistencies in the findings of previous studies and the importance of evaluating visual quality with contact lenses in various light conditions (especially in young individuals who are the main users of contact lenses), this study was designed to investigate the changes in CS resulting from wearing clear and colored hydrogel contact lenses, as the most common types of contact lens fitted, under different light conditions.

##  METHODS

In this cross-sectional study, 34 medical students were recruited from Zahedan University of Medical Sciences. At the outset, a questionnaire was used to screen the general health of the participants and complete ocular examinations were performed. Individuals with systemic diseases, eye diseases such as dry eye, glaucoma, opacity in the ocular media, any obvious ocular abnormality, and a history of trauma and eye surgery were excluded from the study. Refractive errors were determined using Auto Refractometer (AR 8800, Topcon, Japan). Individuals with an uncorrected visual acuity of 6/6 were enrolled in the study. The mean refractive error of the subjects was 0.20 ± 0.46 D (–0.5 to +1.25). Moreover, the subjects had an astigmatism below 0.5 D. Visual acuity was measured using a tumbling E chart at a distance of 6 m, and the Pelli Robson chart was positioned at a distance of 1 m to assess CS. This chart consists of eight rows and each one comprises two separate three-letter columns, with the highest contrast represented in the highest left row and the lowest contrast shown in the lowest row, while the right side of the chart has a log contrast equal to 2.25. The criterion for recording the log CS was reading at least two letters from each three-letter segment.^[19]^ These tests were also recorded in mesopic (3 lux) and glare (2000 lux) conditions, where illumination was measured with TES 1337 B photometer (ES Electrical Electronic Corp. Taiwan). A 60 w tungsten lamp was placed at a distance of 18 cm from the patient's eye and 2 cm above the patient's head (an angle of 10º to the line of sight) in order to create glare. It should be noted that patients were exposed to each lighting condition for at least 20 min in order to make the eyes adapt to light. Then, visual acuity and CS were measured. In each lighting condition, pupil size was measured using a hemispherical scale ruler. The sequence of lighting conditions was chosen randomly.

While the most fitted contact lenses are hydrogel contact lenses, in this study a clear hydrogel contact lens [Bausch & Lomb (Soflens), overall diameter of 14.0, Bace curve radius (BCR) of 8.7, water content of 38.6%] was applied to one eye, and a colored contact lens (Clearcolor, overall diameter of 14.5, BCR of 8.6, water content of 42%, and pigment-free optical zone of 6 ml) to the other eye. In order to reduce the effect of eye laterality on CS, the patient's eyes were randomly selected to fit colored and clear lenses. After 30 min, the fit, movements, cornea coverage, and centration of the contact lenses were evaluated. In case of appropriate fitting, visual acuity and CS tests were measured after 2 hr for each eye under different lighting conditions.

### Data Analysis

Repeated measures ANOVA was employed to analyze the effect of contact lenses on CS under different lighting conditions. The results of pairwise comparison with Bonferroni correction were expressed as the mean and 95% confidence interval of differences. *P* < 0.05 were considered statistically significant.

### Ethical Considerations

All procedures performed in studies involving human participants were in accordance with the ethical standards of the institutional and/or national research committee and with the Helsinki Declaration and its later amendments. Approval was obtained from the Ethics Committee of Zahedan University of Medical Sciences (Ethics approval number: IR.ZAUMS.REC.1399.003). Informed consent was obtained from all participants.

##  RESULTS

Thirty-four students (14 females and 20 males) aged 19 to 23 years (mean, 21.29 ± 1.08 years) participated in this research. The pupil size in right and left eyes was similar in all participants. The mean and standard deviation of pupil diameters were 3.03 ± 1.09 in normal light condition, 5.28 ± 0.94 in mesopic condition, and 1.56 ± 0.59 in glare condition, indicating significant variations in the three lighting conditions (*P* < 0.001). The visual acuity of all subjects was 20/20 which did not change under different light conditions with and without contact lenses. The mean scores of CS in different light conditions with and without contact lenses are given in Table 1. Repeated measures ANOVA displayed the significant impact of contact lenses on CS (*P* < 0.001).

**Table 1:** Mean and standard deviation of log contrast sensitivity under different lighting conditions in clear and colored contact lenses


	**Normal light condition**	**Glare condition**	**Mesopic light condition**
Without CL	1.81 ± 0.11	1.69 ± 0.09	1.51 ± 0.10
Clear CL	1.74 ± 0.10	1.65 ± 0.12	1.46 ± 0.07
Tinted CL	1.74 ± 0.12	1.64 ± 011	1.46 ± 0.12
CL, contact lens			

Furthermore, pairwise comparison showed a decrease in CS when contact lenses were applied compared to the situation where no contact lens was used (mean difference of CS; without CL and with clear CL: 0.031, 95% CI: 0.008–0.053; *P *= 0.005, without CL and with colored CL: 0.029, 95% CI: 0.007–0.052; *P *= 0.007). Meanwhile, the change in CS was not significantly different in the two types of contact lenses [clear and colored (*P* > 0.99)].

In addition, the effect of lighting conditions on CS was significant (*P* < 0.001) with CS under mesopic and glare conditions lower than that under normal lighting condition; more specifically, CS was lower under mesopic condition than that under glare condition (mean difference of CS; between normal light condition and mesopic condition: 0.325, 95% CI: 0.290–0.360; *P* < 0.001, between normal and glare condition: 0.108, 95% CI: 0.058–0.159; *P* < 0.001, between glare and mesopic condition: 0.217, 95% CI: 0.168–0.266; *P* < 0.001).

Moreover, no significant interaction was found between contact lenses and various lighting conditions (*P *= 0.227). Hence, it could be inferred that the effect of contact lenses on reducing CS is similar under different lighting conditions.

**Figure 1:**
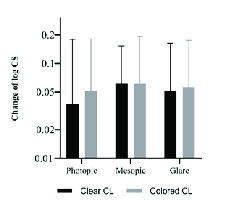
Change in contrast sensitivity (contrast sensitivity without contact lenses minus contrast sensitivity with clear/colored contact lenses) under three lighting conditions.

To further explore the impact of contact lenses in various lighting condition, we studied the change in CS due to contact lenses (CS without contact lenses minus CS with contact lenses) under three different lighting conditions for each participant [Figure 1]. The results of ANOVA test exhibited no significant disparity in CS difference under three lighting conditions (*P *= 0.829).

##  DISCUSSION

Since CS tests provide a better assessment of the quality of vision, the present study investigated CS under different lighting conditions in clear and colored contact lenses. The results proposed that these contact lenses reduce CS but do not affect visual acuity. This CS reduction was similar in both clear and colored contact lenses.

Consistent with our results, Briggs reported a decline in CS as a result of wearing colored and clear contact lenses and similarly found no difference in CS between the two types of contact lenses.^[20]^ Another research showed that clear contact lenses, as opposed to colored contact lenses, did not reduce CS.^[16]^ The greater corneal edema in colored contact lenses compared to clear lenses was not a possible cause in their study and it was stated that the main reason for this difference was the lower optical quality of colored lenses compared to clear ones.^[16]^ Other studies suggested that the diameter of pigment-free optical zone in colored contact lenses plays a substantial role in aberrations, visual acuity, and CS; aberrations and CS in colored contact lenses with an optical diameter of 6 mm do not differ from those of clear contact lenses. However, CS decline at spatial frequency of 12 cycles per degree under photopic condition has been more intense in colored contact lenses with an optical diameter of 4–5 mm than in clear contact lenses.^[18]^

Previous studies have attributed the drop in CS in soft contact lenses to the lack of astigmatism correction, lens deposition, and corneal edema secondary to reduced oxygen supply caused by the contact lens.^[20,21]^ In the present study, the patients had 6/6 visual acuity without optical correction and the power of contact lenses used were plano; therefore the lack of astigmatism correction or spherical aberration cannot explain the decrease in CS. Besides, since the contact lenses were disposable, deposition had no role in diminishing CS. We assessed CS 2 hr after contact lenses were worn. Previous studies have indicated that corneal edema occurs even 1 hr after wearing contact lenses.^[22]^ Hence, the most likely explanation for the decrease in CS in our study may be corneal edema resulting from contact lens application.

However, one of the limitations of our study was that we did not measure corneal thickness before and after 2 hr of lens wearing. It would have been better to measure the thickness of cornea in order to understand corneal edema more accurately. In addition, similarity in CS reduction in the two types of clear and colored contact lenses in this study could be due to the large diameter of the pigment-free optical zone in the colored contact lens (6 mm), which is in keeping with the study by Jung et al.^[18]^ This finding supports the ineffectiveness of optical parameters of clear and colored plano contact lenses and, on the other hand, reinforces the active role of corneal edema in CS reduction.

The present study revealed that visual acuity does not change under different lighting conditions with and without contact lenses. Our results showed that both glare and mesopic conditions led to a CS lower than that obtained by normal lighting conditions. Moreover, CS was lower under mesopic condition than under glare condition. Additionally, both types of contact lenses caused approximately equal amount of reduction in CS under different lighting conditions. Speraul observed that soft contact lenses diminish visual performance in both glare and mesopic conditions.^[23]^ Another study concluded that contact lens could heighten glare sensitivity if it causes corneal epithelial edema; alternatively, no change in glare sensitivity would occur if this edema does not emerge.^[24]^ This unchanged glare sensitivity has been noted in colored contact lenses as well.^[25]^ Another study showed that while soft contact lenses do not affect glare disability, they can induce glare effects.^[26]^

Since placing an additional optical surface such as a contact lens can augment the amount of light scattering and absorption, it is plausible to observe a decline in CS under different lighting conditions when contact lenses are worn. Moreover, the reason for the sharper drop in CS under mesopic conditions can be explained by the development of mydriasis. This dilation of the pupil, in addition to increasing aberrations, can cause flare and further reduce CS when contact lenses are applied. This is due to the fact that pupil diameter exceeds the diameter of the optical zone of the lens.

Our results revealed no significant difference in CS reduction under three lighting conditions using two types of clear and colored contact lenses. This could be related to the type of test used to measure CS. In fact, Pelli Robson only evaluates CS at low spatial frequencies (one cycle per degree), and it fails to assess visual performance at mid and high spatial frequencies. It should be reminded that previous studies have observed the decline in CS only at high spatial frequencies with contact lenses.^[17,18]^ It is recommended that future researchers explore CS by other tests which cover a wider range of spatial frequencies. Furthermore, it is better to compare the CS reduction in various types of contact lenses in the future studies.

In summary, this study indicated that clear and colored contact lenses reduce CS. Since glare and mesopic lighting conditions can also reduce CS, the effect of contact lenses on critical visual functions should be considered in real-world tasks such as driving at night or in the fog.

##  Financial Support and Sponsorship

This study was funded by Zahedan University of Medical Sciences (Grant number: IR.ZAUMS.REC.1399.003).

##  Conflicts of Interest

The authors declare that they have no conflict of interest.
